# Greater resting state functional connectivity of the medial prefrontal cortex with the thalamus, caudate, and putamen in individuals who adhere to the Mediterranean style diets

**DOI:** 10.1007/s00394-024-03548-y

**Published:** 2024-11-28

**Authors:** Paul Faulkner, Paul Allen, Adele Costabile, Marieke H. Schoemaker, Florencia Imakulata, Piril Hepsomali

**Affiliations:** 1 School of Psychology, University of Roehampton, London, SW155 4JD UK; 2 Department of Neuroimaging, Institute of Psychology and Neuroscience, Kings College London, London, UK; 3 School of Life and Health Sciences, University of Roehampton, London, SW155 4JD UK; 4 FrieslandCampina, Amersfoort, the Netherlands; 5 School of Psychology and Clinical Language Sciences, University of Reading, Reading, RG6 6ET UK

**Keywords:** Mediterranean diet, Diet quality, Prefrontal cortex, Thalamus, Caudate, Putamen

## Abstract

**Purpose:**

Healthy diets are believed to be associated with a reduced risk of experiencing common mental disorders (CMDs) and related symptomatology (such as ruminative thinking), and with healthier brain chemistry and structure, especially in the frontal regions implicated in CMDs, cognitive control, and food choice. Nevertheless, there is very limited research on the relationship between diet health/quality and brain function. In this study we assessed the associations between adherence to the Mediterranean diet and resting state functional connectivity (rs-FC) of the prefrontal cortex (PFC) with the whole brain and whether this connectivity would be associated with ruminative thinking as a transdiagnostic factor for CMDs.

**Methods:**

Thirty-seven adults (Mean Age = 25.57, SD = 7.18) completed the Mediterranean Diet Adherence Screener (MEDAS) and were classified into high- and low-quality diet groups and completed the Ruminative Response Scale. All participants underwent resting-state functional MRI (fMRI) to determine whole-brain rs-FC of the medial prefrontal cortex (mPFC).

**Results:**

Participants in the high MEDAS group (vs. low MEDAS group) exhibited significantly greater rs-FC of the mPFC seed with the thalamus, caudate and putamen. Additionally, the strength of rs-FC of the mPFC seed with these regions was positively associated with the MEDAS scores across groups in both crude and adjusted models. There were no significant associations between the strength of rs-FC of the mPFC seed with the cluster of voxels with the thalamus, caudate, and putamen and ruminative thinking.

**Discussion:**

This work shows that healthy dietary patterns are associated with rs-FC in the frontal-subcortical circuitry in healthy volunteers. Considering the implications of the dysregulation of this circuity, adhering to healthy dietary patterns may offer a promising alternative/complementary method to improve CMDs, cognitive control, and food choices.

## Introduction

A growing body of research suggests that several healthy dietary patterns such as the Mediterranean diet (MED), as well as individual food types (e.g., fruits and vegetables) and nutrients (e.g., dietary fibre) that comprise the MED, have been shown to be associated with a lower likelihood of experiencing common mental disorders (CMDs) such as depression and anxiety, as well as CMD-related symptomatology [1–5]. Additionally, data from multiple randomised clinical trials have also demonstrated a causal effect of the healthy dietary patterns improving these outcomes [6–9], possibly via modulation of various mechanisms including, but not limited to, oxidative stress, plasticity, microbiota–gut–brain axis, and inflammatory responses [10, 11].

Nonetheless, there is limited research on the relationship between diet and brain imaging biomarkers. Studies focusing on dietary patterns showed that the MED is associated with structural alterations in the brain, such as greater total brain volume [12, 13], greater grey matter volume in the frontal lobes [13, 14] and preserved integrity of white matter bundles linking limbic regions with the frontal regions [15].

Recently, work from our group has shown that adherence to the MED was associated with increased gamma-aminobutyric acid (GABA) and reduced glutamate (GLU) concentrations, as well as increased grey matter volume in the areas in the prefrontal cortex (PFC; an area that is involved in the aetiology and maintenance of CMD [14, 16–19] and regulation of food intake (type, quantity, and quality) and appetite [20]), and these neural changes were associated with rumination (a transdiagnostic factor for CMDs) [14]. Importantly, the PFC is structurally and functionally connected to the thalamus and striatal regions [21] which are involved in food choice, reward, and motivation [22]. Because there may be a bidirectional relationship between making healthy dietary choices and/or adhering to MED style diets and better CMD outcomes, it may be that stronger resting state functional connectivity (rs-FC) of the PFC with the thalamus and/or striatum is associated with a greater adherence to the MED diet.

Converging evidence from a health neuroscience framework of obesity/overeating highlighted that reduced PFC activity could be viewed as a predictor, cause, or outcome of poor dietary choices/adhering to unhealthy dietary patterns, through downregulation of the PFC (i.e., poor cognitive control, hence dietary self-regulation and food choice) and upregulation of the striatum (i.e., increased reward sensitivity) [23]. Similar to this model, diet-related neurochemical and neurostructural changes in the PFC might, therefore, alter the functioning of the PFC, thalamus, and striatum. This may offer an additional mechanistic explanation as to why there may be a bidirectional relationship between making healthy dietary choices and/or following the MED style diets and better CMD outcomes. However, apart from a small number of studies that identified (i) localised changes in functional brain activity in response to intake of specific nutrients [24] (which fails to account for the synergistic and cumulative effects of multiple nutrients within a comprehensive dietary pattern [25]), and (ii) increased within-network and between-network resting-state functional connectivity (rs-FC) in individuals who adhere to the MED [26], and reduced rs-FC in the structures that are involved in pathophysiology of obesity/overeating [27], no studies have investigated the associations between MED and rs-FC of the prefrontal cortex. To this end, we performed a seed-based analysis to assess the associations between adherence to healthy eating patterns (i.e., the MED), resting-state functional connectivity of the PFC, and the CMD transdiagnostic factor, rumination. We hypothesised that connectivity of the PFC with those regions identified as important for CMDs, food choice, reward, and motivation would be weaker in individuals with low adherence to the Mediterranean diet, and that this connectivity would be associated with rumination.

## Methods

### Participants

One hundred and sixty-four students (from the University of Roehampton and University of Royal Holloway) and members of the public were screened online (Qualtrics; https://www.qualtrics.com) using the Mediterranean Diet Adherence Screener (MEDAS) [28]. Thirty-eight participants were selected based on the upper and lower quartiles to establish high MEDAS score (High MEDAS; >8, *n* = 19) and low MEDAS score (Low MEDAS; <6, *n* = 19) groups.

Exclusion criteria included presence of contraindications for MRI scanning (i.e., presence of metal, etc.), current use of prescribed medication for neuropsychiatric disorders or illicit substances misuse, or history of or presence of psychiatric and neurological disorders, body mass index < 18.5 kg/m^2^ and > 29.9 kg/m^2^, and having diabetes mellitus, hypertension (systolic blood pressure ≥ 140 mmHg or diastolic blood pressure ≥ 90 mmHg) or cardiovascular diseases (clinical history). All participants provided informed consent and received £50 for participation. The research protocol was approved by the Ethical committee at the University of Roehampton (Reference: PSYC 22/ 444) on 30/01/2023. The study has been performed in accordance with the ethical standards laid down in the 1964 Declaration of Helsinki and its later amendments.

### Demographic, dietary, and clinical assessment

In order to ensure that High vs. Low MEDAS groups were matched for demographic and environmental/lifestyle factors, all participants were asked to complete a demographics form assessing age, sex, level of education, handedness (assessed via Annett Hand Preference Questionnaire [29]), income, alcohol consumption (units per day), and tobacco consumption (cigarettes per day)…etc. Additionally, EPIC Norfolk Food Frequency Questionnaire (FFQ) was used [30] to estimate habitual food intake. Participants reported (i) how frequently they consume 130 food items, with options ranging from ‘never or less than once a month’ to ‘6 + per day’ and (ii) other foods they consume. FETA software was used to convert food frequency questionnaire data into nutrient and food group values [31].

A-14-item MEDAS [28] was used to estimate adherence to the Mediterranean style diets. This measure involves 12 questions on food consumption frequency (e.g., “How many servings of whole fruit do you eat per day?”) and 2 questions on food intake habit (e.g., “Do you use olive oil as the main source of fat for cooking?”). Each question is scored 0 or 1, and total scores range from 0 to 14 where higher scores represent higher adherence to the Mediterranean diet, hence higher diet quality. The MEDAS demonstrates good test-retest reliability (*r* = 0.69) [32] and validity (coefficients ranging from 0.52 to 0.79) [28, 32, 33] in healthy and unhealthy adult populations in the UK and in other European countries.

Ruminative Response Scale (RRS) [34], a 22-item questionnaire, was used to assesses reflection, brooding, and depression-related rumination. Each item (e.g., “How often do you Think about how sad you feel?”) is rated on a 5-point Likert scale from 1 (almost never) to 4 (almost always) and higher scores reflect increased levels of ruminative thinking. The RRS demonstrates good test-retest reliability (*r* = 0.67) and validity (0.90) [34].

High and Low MEDAS groups were compared on demographic and clinical measures by using chi-square or independent sample t-tests (two-tailed) on IBM^®^ SPSS Statistics Version 26. A threshold of *p* < 0.05 was applied throughout.

### fMRI data acquisition

Resting-state fMRI images were acquired over 10 min using a 3-T Siemens AG Trio MRI system with a 32-channel head coil while participants were instructed to keep their eyes closed (300 T2*-weighted echoplanar images; repetition time = 2 s; echo time = 30 milliseconds; slice thickness = 4 mm; flip angle = 90°; matrix: 64 × 64; field of view = 192 mm). A T1-weighted magnetization-prepared rapid-acquisition gradient echo (MPRAGE) scan was acquired for registration purposes, including spatial normalization to standard space (Montreal Neurological Institute; MNI).

### fMRI data preprocessing

Image analysis was performed using FMRIB Software Library (FSL) version 6.0.6 (www.fmrib.ox.ac.uk/fsl). The time course of the fMRI data was first realigned to compensate for head movements as in Jenkinson, et al. [35]Jenkinson, et al. [35], and all non-brain matter was removed using FSL’s brain extraction tool (BET). Time-series statistical analysis was carried out using FSL’s Improved Linear Model, with local autocorrelation correction as in [36], after high-pass temporal filtering (Gaussian-weighted least square fit (LSF) straight line fitting with sigma = 50 s).

None of the fMRI data were deemed to have had excessive head motion (> 2.5 mm translation). Motion cleaning and noise reduction were performed using a 32-parameter linear regression model [37] used in our previous publications [38–40]. Specifically, this included 6 motion parameters (3 translational dimensions along the X, Y and Z axes as well as the 3 rotational dimensions of ‘pitch’, ‘roll’ and ‘yaw’) combined with the timeseries from the CSF and white matter (all of which provides 8 parameters), as well as their temporal derivatives to provide 16 parameters, and the quadratic of these to provide 32 parameters in total. Furthermore, frame-wise displacement (FD) was determined with root-mean squared matrix calculation (using the tool ‘fsl_motion_outliers’) to obtain the average rotation and translation parameter differences across EPIs. Time points where motion exceeded acceptable FD thresholds as expressed in [41, 42] were censored using separate regressors for each of these time points in the model. A fixed FD threshold for all participants was determined via calculation of the SD of FD across all data points and computing the following equation: 0.25 mm + 2 * SD as in Satterthwaite, Elliott, Gerraty, Ruparel, Loughead, Calkins, Eickhoff, Hakonarson, Gur, Gur and Wolf [37]Satterthwaite, Elliott, Gerraty, Ruparel, Loughead, Calkins, Eickhoff, Hakonarson, Gur, Gur and Wolf [37]. The timeseries of the resultant residuals from the regression model was then scaled and normalised at each voxel: ([residuals – mean]/SD) + 100.

**Fig. 1:**
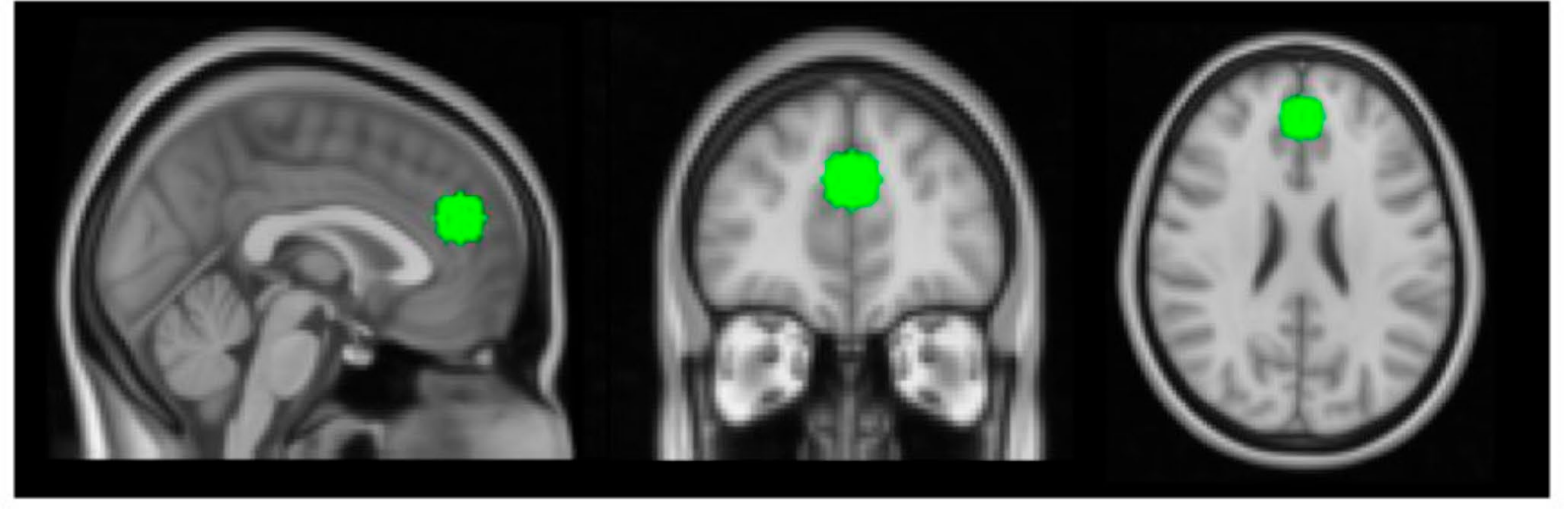
Placement of seed in the mPFC in standard (MNI) space

A seed-based approach was implemented by creating a 10 mm sphere in the mPFC with a centre-of-mass (in MNI space) of X = 45, Y = 85 and Z = 48 (Fig. 1). This placement of the seed was chosen to mirror the placement of the voxel for the ^1^H-Magnetic Resonance Spectroscopy scans performed on the same participants as shown in our previous paper [14]. This seed mask was transformed to each participant’s native space, and the timeseries data from within this mask were then extracted from the scaled and normalised residuals of the 32-parameter regression model and then included as a single explanatory variable in a linear model at the first (i.e., single-subject) level. Contrast images representing voxel-wise effects resulting from this model were then registered to the MPRAGE structural image and then into standard MNI space using a 12-paramater affine transformation [43]. Registration from MPRAGE structural images into standard space was further refined using nonlinear registration [i.e. FNIRT; 44]. Finally, images were smoothed using a 5 mm full-width at half-maximum Gaussian kernel.

### fMRI data analysis

Group level analyses were performed using FSL’s Local Analysis of Mixed Effects (FLAME1) with outlier de-weighting applied.

To assess the influence of group (High vs. Low MEDAS) on whole-brain connectivity of the mPFC seed, a model with five explanatory variables (EVs) that controlled for BMI, age and sex was constructed. The first EV denoted the High MEDAS group, the second EV denoted the Low MEDAS group, the third EV denoted BMI (demeaned), the fourth EV denoted age (demeaned), and the fifth EV denoted sex (demeaned). Two contrasts were computed in order to determine which in regions (if any) (i) the high MEDAS group exhibited greater connectivity with the mPFC seed and (ii) the low MEDAS group exhibited greater connectivity with the same mPFC seed.

To assess the relationship between scores on the MEDAS and whole-brain connectivity of the mPFC seed, a model with four explanatory variables (EVs) that controlled for BMI, age and sex was constructed. In this model, the first EV denoted MEDAS scores, the second EV denoted BMI, the third EV denoted age, and the fourth EV denoted sex (all demeaned). Two contrasts were computed to determine which regions (if any) exhibited resting-state functional connectivity that (i) positively correlated and (ii) negative correlated with MEDAS scores in the whole group.

For all models, statistical maps were cluster-corrected for multiple comparisons (voxel height threshold: *Z* > 3.09, cluster significance *p* < 0.05).

To examine the relationships of rumination with group differences in connectivity, connectivity values (z scores) were extracted from significant clusters and entered into the IBM^®^ SPSS Statistics Version 26 for correlation with the RRS.

## Results

### Participant characteristics

Due to data loss, we report results from 37 participants (19 High MEDAS and 18 Low MEDAS). Table 1 provides a full summary of participant characteristics across the sample and in High MEDAS and Low MEDAS groups. The groups did not differ for sex, age, education, income, physical activity, BMI, energy and macronutrient intakes, and handedness, but by design, differed significantly on the MEDAS scores.

**Table 1:** Participant characteristic across the sample and in the high MEDAS and low MEDAS groups

	Total Sample (*n* = 37)	High MEDAS (*n* = 19)	Low MEDAS (*n* = 18)	t/χ2	*p*
**Sex** (N: M/F)	13/24	7/12	6/12	0.05	0.82
**Age** (M ± SD)	25.57 ± 7.18	26.21 ± 8.71	24.89 ± 5.28	− 0.55	0.58
**MEDAS Score** (M ± SD)	6.92 ± 2.30	8.84 ± 0.90	4.89 ± 1.37	-10.45	**< 0.001**
**RRS Score** (M ± SD)	46.46 ± 14.66	46.58 ± 15.25	46.33 ± 14.45	0.05	0.96
**Education** (N)				3.88	0.42
* GCSE/O levels*	1	0	1		
* A levels/secondary*	8	5	3		
* Degree commenced*	6	2	4		
* Degree completed*	12	8	4		
* Postgraduate*	10	4	6		
**Income/annum** (N)				1.82	0.87
* Less than £18*,*000*	7	4	3		
* £18*, *000 to £30*, *999*	8	4	4		
* £31*, *000 to £51*, *999*	8	5	3		
* £52*, *000 to £100*, *000*	3	2	1		
* More than £100*, *000*	5	2	3		
* Do not know*	6	2	4		
**Physical Activity/week** (N)				2.67	0.62
* Less than 30 min*	3	1	2		
* 30–90 min*	6	3	3		
* 90–150 min*	13	8	5		
* 150–300 min*	9	3	6		
* More than 300 min*	6	4	2		
**BMI** (kg/m²) (M ± SD)	24.37 ± 4.69	23.53 ± 4.35	25.26 ± 5.00	-1.12	0.27
**Energy/day** (kcal) (M ± SD)	1738.09 ± 637.26	1764.12 ± 688.43	1710.61 ± 597.17	0.25	0.80
**Carbohydrate/day** (g) (M ± SD)	201.26 ± 82.25	199.09 ± 88.63	204.79 ± 77.39	− 0.21	0.84
**Protein/day** (g) (M ± SD)	85.65 ± 32.81	85.26 ± 36.66	86.14 ± 29.27	− 0.08	0.94
**Fat/day** (g) (M ± SD)	67.00 ± 28.44	71.34 ± 30.36	64.46 ± 26.68	0.73	0.47
**Handedness** (N: R/L)	34/3	18/1	16/2	0.42	0.52

N: number; M: male; F: female; MEDAS: Mediterranean Diet Adherence Screener; RRS: Ruminative Response Scale; kg: kilograms; m: metre; kcal: kilocalories; g: grams; R: right; L: left

### Resting state functional connectivity

**Fig. 2:**
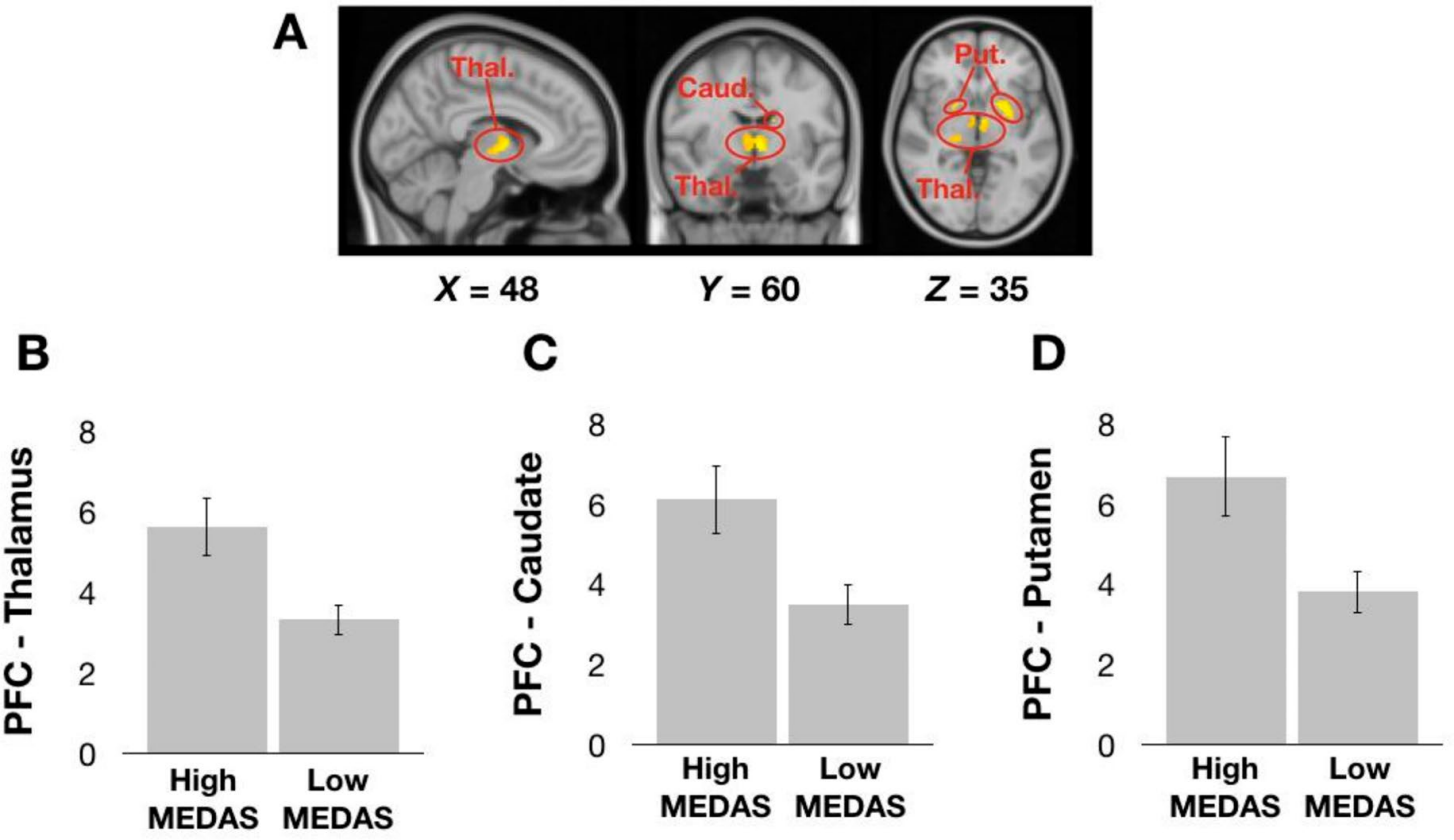
**A**: Cluster of voxels in which resting-state functional connectivity with the mPFC seed depicted in Fig. 1 is greater in the High MEDAS group than in the Low MEDAS group. **B-D**: Mean z values from the cluster of voxels in the Thalamus (**B**), Caudate (**C**) and Putamen (**D**) in High MEDAS individuals and Low MEDAS individuals separately. Thal. = Thalamus; Caud. = Caudate; Put. = Putamen. Error bars denote 1 standard error of the mean

Whole-brain, voxel-wise GLM analyses revealed that compared to individuals in the Low MEDAS group, those in the High MEDAS group exhibited greater resting-state functional connectivity of the mPFC seed with the thalamus, caudate and putamen (Fig. 2). We did not observe any significant clusters for Low MEDAS > High MEDAS contrast.

**Fig. 3:**
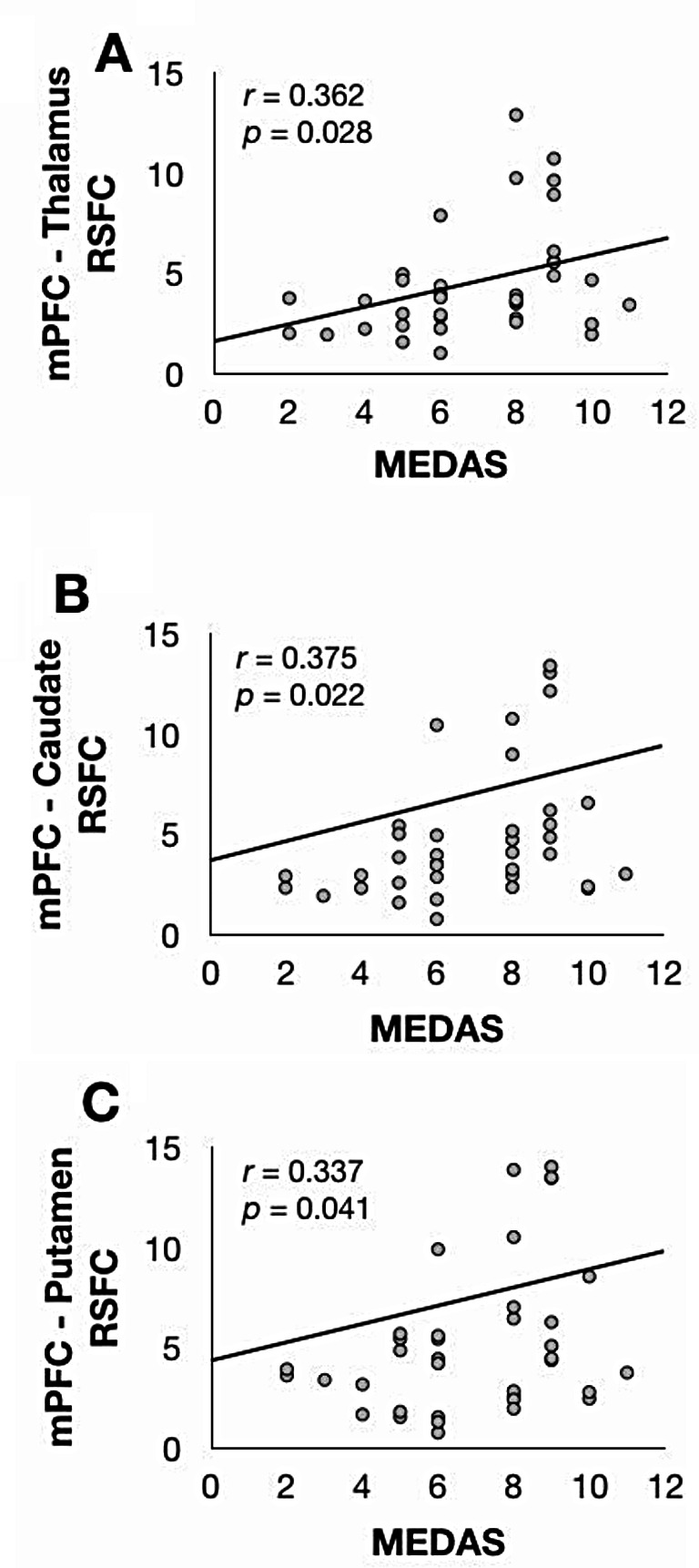
Scatterplots depicting positive bivariate correlations between MEDAS scores and z scores extracted from within the **A**: Thalamus; **B**: Caudate; and **C**: Putamen clusters depicted in Fig. 2 above

The GLM that examined the relationship between whole-brain connectivity of the mPFC seed and continuous MEDAS scores in the whole group revealed no significant clusters. As such, we extracted the values from each of the three separate clusters depicted in Fig. 2 and correlated these with MEDAS scores in all participants in an exploratory fashion. Bivariate correlations revealed that, in the whole group, MEDAS scores positively correlated with resting-state functional connectivity of the mPFC seed with the cluster of voxels with the thalamus (*r* = 0.362, *p* = 0.028), caudate (*r* = 0.375, *p* = 0.022) and putamen (*r* = 0.337, *p* = 0.041) (Fig. 3). Partial correlations revealed that these correlations remained significant when controlling for the effects of BMI, age, and sex revealed (thalamus: *r* = 0.398, *p* = 0.020, caudate: *r* = 0.377, *p* = 0.028; putamen: *r* = 0.346, *p* = 0.045).

Bivariate and partial correlations did not reveal significant associations between RRS (i.e. rumination scores) and resting-state functional connectivity of the mPFC seed with the cluster of voxels with the thalamus, caudate, and putamen.

## Discussion

The aim of the current study was to perform a seed-based analysis to assess the associations between adherence to healthy eating patterns (i.e., the MED) and rs-FC of the PFC with those regions identified as important for CMDs, food choice, reward, and motivation. Results revealed greater rs-FC of the mPFC seed with the thalamus, caudate and putamen in individuals in the high (vs. low) MEDAS groups. Additionally, rs-FC of the mPFC seed with the cluster of voxels with the thalamus, caudate, and putamen were positively associated with the MEDAS scores across groups in both crude and adjusted models. However, rs-FC of the PFC with thalamus, caudate, and putamen were not significantly associated with the RRS scores.

Our study provides the first direct evidence showing greater rs-FC of the mPFC seed with the striatal regions (including caudate and putamen) and thalamus in individuals in the high (vs. low) MEDAS groups. Additionally, rs-FC of the mPFC was positively correlated with MEDAS scores across both groups. Previous research showed that weaker PFC activity is implicated in impairments in cognitive control [45, 46] (which is linked to the aetiology of CMDS [47]), possibly through modulation of GABAergic, glutamatergic, and dopaminergic neurotransmission/metabolism in this area [18, 48–50]. Increased striatal/thalamic activity, on the other hand, is known to be involved in impairments in impulsivity/compulsivity [51, 52], reward [53], as well as motivation [22], and intake of palatable calorie-dense foods increases striatal cortical activity [54], possibly through decreased dopaminergic signalling/activity, as previously observed in the forms of (i) decreased binding potential for dopamine D2 receptors in obese humans [55] (as a consequence of insulin resistance [56]) and decreased dopamine D2 receptors in the striatum in rats who were fed high fat (vs. normal chow) diets [57, 58] and/or (ii) increased microglia proliferation, hence, metabolic inflammation following high-fat diet consumption [52]. As depicted in models of obesity/over-eating, downregulation of PFC and upregulation of striatum is implicated in impairments in food choice-related self-control and cognitive control, which may increase tendency to consume unhealthy foods/follow unhealthy dietary patterns [59–61]. Additionally, PFC-thalamus connectivity is an integral part of decision-making [62] and external eating behaviours (i.e., eating in response to food cues) [63]. Taken together, adherence to unhealthy dietary patterns may impact the functional calibration of this frontal–subcortical circuitry, by evoking functional dysregulation of the PFC and striatal and thalamic regions (potentially through diet-induced alterations in neurotransmission/metabolism of GABA/GLU/Dopamine). In turn, this may result in potential impairments in cognitive control and thus in experiencing symptoms of CMDs, and/or adherence to unhealthy dietary patterns/making poor food choices through altered impulsivity, motivation, and reward processing. Therefore, dietary approaches that alter frontal and striatal GABA, GLU, and dopamine neurotransmission/metabolism, may offer a promising approach to recalibrate this frontal–subcortical circuitry, and potentially improve CMDs and CMD-related symptomatology and/or making healthy food choices. This may be true in part due to the fact that adherence to low GLU diets and consumption of high dietary GABA have already been shown to affect brain and behaviour (improved subjective stress and sleep-related outcomes and altered cognitive performance, blood oxygen level dependent (BOLD) response and functional connectivity) [3, 64, 65], possibly through GABA-modulating bacteria of the human gut microbiota [66].

The MED emphasises food groups intrinsically related to brain health (hence potentially to better affective and cognitive processing and outcomes) and is characterised by high intakes of vegetables, fruits and nuts, legumes, and unprocessed cereals, low intakes of meat and meat products [67] that are associated with various metabolic processes [10, 11]. Hence, the MED may also potentially alter neuro-chemistry,-structure, and/or -function, and related affective and cognitive mechanisms through inhibition of oxidisation and inflammatory pathways [68, 69] and through modulating peripheral and central glucose and insulin metabolism [68, 70] and the gut microbiota [71–73].

Although in our previous study we observed a significant negative correlation between rumination and rPCG-GMV and a marginally significant positive association between rumination and mPFC-GLU concentrations [14], in the current study rs-FC of the mPFC with the striatal and thalamic regions were not correlated with RRS scores. Given that our measure of rumination in the current study was based on self-report, the lack of correlations may reflect diminished insight into ruminative-thinking processes driven by lower regional connectivity in the frontal and higher regional connectivity the striatal areas that were reported to be associated with more self-reported unwanted thoughts [74].

There are several notable caveats to be aware of in this study. Firstly, due to the cross-sectional nature of our study, we could not determine cause and effect relationships, therefore, further longitudinal studies are warranted to allow stronger causal inferences to examine the impact of diet quality on rs-FC. Secondly, the small sample may have led to Type II errors, especially when examining associations of connectivity with RRS scores. Lastly, due to small sample size, we could not examine potential sex-dependent effects, however, future studies are encouraged to consider stratification of results/analysis by sex in order to fully elucidate sexual dimorphism in the context of nutrition and health.

In conclusion, our findings suggest that adhering to healthy dietary patterns may be associated with stronger rs-FC between specific brain structures involved in CMD physiopathology and food choice, reward, and motivation, and may therefore, offer a promising alternative and/or complementary method to improve CMDs and CMD-related symptomatology and/or making further healthy food choices. Considering the potential implications of the PFC dysregulation discussed above, and that (i) the PFC follows a protracted development [75], and (ii) overall brain health may encompass a wide range of adverse outcomes including dementia and functional impairment, the timing of preventative interventions may be especially pertinent.

## Data Availability

The data presented in this study are available on request from the corresponding author.
